# Asylum seeking and refugee adolescents’ mental health service use and help-seeking patterns: a mixed-methods study

**DOI:** 10.1038/s44184-022-00019-2

**Published:** 2022-11-01

**Authors:** Yudit Namer, Alexandra Freţian, Diana Podar, Oliver Razum

**Affiliations:** Department of Epidemiology and International Public Health, Bielefeld School of Public Health, Bielefeld University, Bielefeld, Germany

**Keywords:** Psychology, Health care, Health services, Public health

## Abstract

Almost a third of all people who entered Germany to seek protection since 2010 were under the age of 18. Asylum-seeking and refugee (ASR) adolescents in Germany face reduced entitlements to healthcare and experience barriers in accessing mental healthcare, despite documented mental health needs. This mixed-methods study aims to describe the mental health needs and service use of ASR adolescents in Germany and identify the predictors of their help-seeking patterns. Here we report findings of cross-sectional data collected between February 2019 and November 2020 in schools and refugee accommodations in three German federal states. Our subsample consists of ASR between the ages of 11 and 18, coming from Syria, Afghanistan, and Iraq (*N* = 216). Cross-sectional data are supplemented by semi-structured interviews with nine mental health professionals in one region of the study. Our findings reveal an underutilization of mental health services relative to the emotional difficulties reported. Perceived and experienced access barriers, age, and externalizing and internalizing symptoms predict different help-seeking patterns. Psychotherapy-related social resources, as well as reporting of emotional difficulties, are predictors of actual or intended psychotherapeutic service utilization. Based on our quantitative and qualitative findings, we highlight the need for widespread, accessible, and low-threshold mental health initiatives designed to work with ASR adolescents, for additional assistance in navigating the mental healthcare system, as well as for support to important people in ASR adolescents’ lives who fill the gap between mental health needs and accessible mental healthcare services.

## Introduction

Children and adolescents make up 42% of the 82.4 million people worldwide forcibly displaced due to war, persecution, conflict, violence, human rights violations, or impacts of climate change^1^. Forced migration puts children and adolescents under increased threats that intensify common developmental challenges. Before, during, and after their flight, children and adolescents are confronted with disruptions in their education and social lives, cumulative traumatic experiences, dangerous or protracted journeys, complex and uncertain legal immigration processes, and discrimination and exclusion^2–5^.

While psychological distress is a natural response to traumatic events and displaced children and adolescents show remarkable resilience, the burden of these challenges can have a long-lasting negative impact on physical health and well-being^2,6^. Despite high heterogeneity and methodological variability among studies, prevalence estimates suggest that ASR children and adolescents have higher rates of mental health disorders compared to minors without a history of forced migration^2,3,7–9^. A recent systematic review and meta-analysis has confirmed high rates among ASR minors of mental health issues such as depression (14%), anxiety disorders (16%), and post-traumatic stress disorder (PTSD, 23%)^10^, requiring specialized mental healthcare services^3,11^.

At the time of the study, almost a third (31.9%) of all people who entered Germany to seek protection since 2010 were under the age of 18, and almost a fifth (19.8%) were at the age of compulsory schooling (i.e., 6–17)^12^. In Germany, it is a legal requirement for ASR children and adolescents to attend school analogous to citizen children^13^ and 95% of ASR children and adolescents (aged 10–17 years) are enrolled^14^. Although EU law permits a maximum of three-month delay in school enrollment for ASR children and adolescents, in Germany, enrollment can be delayed for six months due to restrictions in reception centers or regional school allocation practices^14,15^. Historical segregation practices, persisting discrimination experienced by marginalized communities, and school closures due to the COVID-19 pandemic disproportionately affected ASR children and adolescents^16^.

Most of the research on ASR children and adolescents in Germany have been conducted with unaccompanied refugee minors (URM). There is robust evidence that URM experience multiple forms of psychological distress and thus an increased need for mental healthcare services in Germany^7,17,18^. URM further experience a postmigration climate that is not fully welcoming despite the predominant “welcome culture” discourse in Germany^19^ and face added challenges during transfer to independent living when they become adults^20^. Few studies have focused on accompanied (i.e., residing with parents) ASR children and adolescents in Germany, and none so far has focused on their mental health-related help-seeking patterns.

Evidence from multiple European countries shows that despite equitable aspirations, ASR and migrants persistently face barriers to accessing adequate health services^4,21^. Persistent inequalities in access to healthcare services are the results of multiple impediments: restrictive legal regulations, language barriers, lack of cultural competence among health providers, discrimination and racism, unfamiliarity with the health system in a new country, and economic barriers, to name a few^4,21–24^.

In Germany, these inequalities are further deepened for ASR by restrictive healthcare entitlement policies which, for the first 18 months after arrival, only cover emergency care, immunizations, treatment for acute and painful conditions, perinatal care, and other potentially “essential” health intervention. The term “essential” has been left open to the interpretation of administrators/professionals (such as social welfare offices) who often lack (mental) healthcare training^25–28^. Depending on the federal state of resettlement, ASR minors and adults need to obtain healthcare vouchers for every medical visit or are provided with electronic health cards (EHC)^28^. However, both access models are distinct to the access provided to regular citizens and other migrant groups, perpetuating or worsening health inequalities^29,30^.

For mental healthcare, additional barriers are present in Germany. Once ASR are included in the regular statutory health insurance, interpreter services during psychotherapy sessions are no longer covered^31^. Less information about the mental healthcare system is available to ASR than to residents in Germany^32^. In one adult ASR group in Germany, receiving psychotherapy was rare despite the need for psychotherapeutic services. Fear of residence consequences, not having a healthcare voucher, lack of familiarity regarding service seeking, fear of exclusion, not being taken seriously, and language barriers were the reasons for not receiving psychotherapeutic treatment^33^. Furthermore, not all psychotherapists in the German healthcare system feel ready to treat ASR; comfort with interpreters and previous experience predict whether psychotherapists feel ready to work with ARS or not^34^.

Studies conducted in Germany further confirm the inequitable access to timely and appropriate (mental) health services despite long-lasting illness, bodily pain^29^, and higher need, especially among URM^7,18,35^. However, actual mental health service utilization is not yet known for ASR minors in Germany. While the connection between the need for services and their use is complex, understanding existing care pathways and help-seeking patterns are crucial starting points in addressing the mental healthcare needs of ASR children and adolescents and taking the necessary steps to ensure equitable care.

Our study is a subproject in the YOURHEALTH consortium, a longitudinal study investigating the mental health of minor refugees living in Germany. Our mixed-method subproject’s aim is to assess ASR minors’ perceptions, knowledge of, and barriers to accessing psychotherapeutic and mental healthcare services in Germany, focusing on the gap between the ASR minors’ needs and the services provided. In this article, we (i) describe ASR adolescents’ mental health needs and psychotherapy utilization, (ii) identify the predictors of their mental health-related help-seeking patterns, and (iii) contextualize mental healthcare provision from mental health professionals’ perspectives. We accordingly report our findings from a mixed-methods study utilizing cross-sectional survey data collected from ASR adolescents and qualitative data from semi-structured interviews with mental health professionals. We conclude with policy recommendations highlighting the need for widespread, accessible, and low-threshold mental health initiatives designed to work with ASR adolescents.

## Methods

### Study design

The study follows a mixed-methods design. We describe ASR adolescents’ mental health indicators and psychotherapeutic service utilization and identify the predictors of mental health-related help-seeking patterns. We use cross-sectional quantitative data and link the common insights that emerged from semi-structured interviews with mental health professionals to contextualize the negotiation of the mental healthcare system.

### Quantitative data collection and analysis

#### Procedure and participants

Cross-sectional baseline data from a longitudinal dataset collected by the YOURHEALTH consortium were utilized in this study^36–38^. Data collection took place between February 2019 and November 2020 in schools and refugee accommodations, located across three federal states in Northern and Southern Germany. ASR minors aged 11–18 years coming from Syria, Afghanistan, and Iraq were the study’s main target group. ASR adolescents who came to Germany after 2015 and spoke one of the target languages (Arabic, Farsi, Sorani, Kurmancî, Pashto, and German) were invited to participate. This occurred through collaboration with educators (in schools) and administrators (in collective accommodations).

To ensure informed consent, we provided participants with informational videos recorded in multiple languages (Arabic, Farsi, Sorani, Kurmancî, Pashto, and German), detailing the study objectives, procedure, voluntary and confidential nature of participation, and anonymity of results. Participation was voluntary and participants could withdraw their consent at any time. Participants under the age of 16 were enrolled following written informed consent from their parents or legal guardians. Participants were rewarded with a 20 Euro voucher, regardless of questionnaire completion. The different stages of this study (piloting, main study, and annotations due to change in procedure) were approved by the ethics committee of Bielefeld University (applications EUB 2018-161, EUB 2018-174, and EUB 2020-039).

Due to the comprehensive length and sensitive nature of the questionnaire, we took multiple steps to ensure that all the measures are accessible and appropriate for all our participants. All the scales used in our study were previously used in adolescent populations (ages 11–18) or were adapted for our sample and translated into the target languages (Arabic, Farsi, Sorani, Kurmancî, and Pashto) and back-translated into German or English (depending on the source language). Based on inputs from cognitive interviews with ASR minors, and multi-lingual research assistants, we offered the questionnaire in native languages, both in writing and as audio translations on tablets, and simplified the language. Furthermore, our multi-lingual research assistants were present for data collection to provide additional support in participants’ native languages. Facilitated by their language skills, 80.6% of participants opted to complete the German version of the questionnaire.

Data were collected via a tablet- (93%) and paper-pencil (7%)-based self-assessment and given the comprehensive length of the questionnaire (more than 400 questions for the collective study), took place on two different occasions when possible (i.e., when the study location could be reached on subsequent days). This was done to ensure that participants would not be overburdened and could stay focused.

Additionally, due to the sensitive and potentially triggering nature of some of the scales, we developed a protocol and training on mental health first aid. All research assistants were trained on how to implement it, however, there was no need to do so throughout the study. After each data collection session, participants received ‘Aid/Support Cards’ with details for mental health services in their respective regions. These services were contacted by researchers beforehand to ensure their accessibility by participants in need.

#### Measures

The variables of interest can be categorized as mental health indicators (reporting of emotional difficulties, internalizing symptoms, and externalizing symptoms) and mental health-related help-seeking variables (help-seeking patterns, perceived and experienced barriers to psychotherapy, satisfaction with psychotherapy and social resources for psychotherapy). We provided age-appropriate definitions for emotional difficulties and psychotherapists at the beginning of the questionnaire. These were checked for cultural appropriateness with the research assistants who translated the items, and for age appropriateness in the cognitive interviews. The measures are summarized in Table 1.

**Table 1:** Summary of measures.

Variable	Question/Scale	Answer options
Reporting of emotional difficulties	Do you think you have emotional difficulties that you need help with?	Yes/No
Internalizing and externalizing symptoms	Hopkins Symptoms Checklist-37 (HSCL-37A)^39^ – assesses trauma-related common mental health disorders; Internalizing symptoms: anxiety (10 items) and depression (15 items); Externalizing symptoms: conduct disorder (5 items), oppositional-defiant disorder (2 items) and substance abuse (5 items).	Four-point Likert scale (1 = not/never, 2 = sometimes, 3 often, 4 = always)
Mental health help-seeking behavior pattern	a) Have you ever talked to anyone about your emotional difficulties? b) Have you ever been to a psychotherapist?	a) No, I have not talked to anyone/ Yes, with a parent or guardian/ Yes, with an older relative (e.g., grandparents, uncle, aunt)/ Yes, with a relative of the same age (e.g., brother, sister, cousin)/ Yes, with a psychotherapist/ Yes, with a doctor/ Yes, with a friend/ Yes, with a teacher/ Yes, with a mentor or religious leader from the community (e.g., imam, priest, mullah)/ Yes, with a lawyer/ Yes, with someone from my country/ Yes, with someone from a refugee center/ Yes, with someone else:_____ b) Yes/No/No, but I intend to see a psychotherapist
Satisfaction with psychotherapy	a) How satisfied are you with the help you received from your psychotherapist? b) Was there an interpreter present during psychotherapy sessions? c) Do you consider that the interpreter had translated well? d) Did you find the interpreter trustworthy? e) Are you still seeing a psychotherapist? f) If no, why not?	a) Very satisfied - somewhat satisfied - not satisfied b) Yes/No c) Yes/No/I don’t know d) Yes/No e) Yes/No f) I did not feel comfortable/I did not always see the same psychotherapist/Friends and/or relatives have discouraged me/A religious leader has suggested other ways of coping/ I feel better/ I don’t think it has helped/ I don’t have time/ I could not afford it/ Going there was complicated/ Other reasons
Barriers to psychotherapy	12 (no experience of psychotherapy)- or 19-item measure (experience of psychotherapy), adapted from Barriers to Mental Health Services Scale-revised (BMHSS-R)^41^	Four-point Likert scale (from 1 = “not at all” to 4 = “a lot”).
Social resources	Do you know someone who can help you find a psychotherapist if you are having emotional difficulties? Has anyone ever told you to go to a psychotherapist?	Yes/No

### Mental health indicators

#### Reporting of emotional difficulties

Participants responded to the question, “Do you think you have emotional difficulties that you need help with?” with either “yes” or “no.”

#### Internalizing and externalizing symptoms

Participants completed the Hopkins Symptom Checklist-37 (HSCL-37A), a self-report measure on internalizing (anxiety and depression) and externalizing symptoms (conduct disorder, oppositional-defiant disorder, substance abuse) that are associated with reactions to trauma and have been previously validated with ASR population^39^. The answer options to the question of whether one has felt or acted in a certain way during the past month are expressed on a four-point Likert scale, ranging from 1 = “never” to 4 = “always”. Scores can be calculated for all 37 items, which sum up to a total score to describe global psychological distress, ranging (from 37 to 148 points) or for each of the subscales (i.e., anxiety, depression, and externalizing symptoms). We calculated the sum score for internalizing (25 items) symptoms subscale (sum of anxiety and depression items), if there were a maximum of two values missing and for the subscale of externalizing symptoms (12 items) when a maximum of one value was missing. The respective missing values are replaced through imputation by calculating the mean value of the existing items and multiplying it by the respective number of items from each subscale. The internal consistency of internalizing symptoms (α = 0.90) was very good, whereas externalizing symptoms (α = 0.65) was moderate, although in line with previous studies for ASR adolescent samples in the Netherlands and Germany^17,18,39^. While the scale has no set clinical cutoff point, we used the ones suggested by previous literature (i.e., a total score of 69 points) derived from percentile scores from research with URM in Belgium^40^.

### Mental health help-seeking related variables

#### Mental health help-seeking pattern

We constructed two mental health-related help-seeking pattern variables for the purposes of different analyses based on the responses to the following questions: “Have you ever talked to anyone about your emotional difficulties?”, where participants could choose multiple conversational partners from contexts within and outside of the mental healthcare system, and “Have you ever been to a psychotherapist?”, where participants could choose between following options: “yes”, “no”, or “no, but I intend to”.

We constructed two binomial variables: (1) seeking help from mental health-related professionals (i.e., went to a doctor, psychotherapist, or social worker) (yes/no) and (2) seeking help from people outside of the mental healthcare system (i.e., talked to their parents, family members, friends, teachers, religious leaders, lawyers, etc. about their emotional difficulties) (yes/no).

#### Satisfaction with psychotherapy

We asked participants with experience in psychotherapy, “How satisfied are you with the help you received from your psychotherapist?” (Answer options: “very satisfied”, “somewhat satisfied”, “not satisfied”). Participants were also asked to report if they had an interpreter present during their psychotherapy sessions (“yes”/“no”), if they interpreted well, and if they were trustworthy (“yes”/“no”/“I don’t know”). Finally, participants were asked if they are still seeing a psychotherapist (“yes/”no”) and if not, to provide the reasons from an answer list (e.g., “I didn’t feel comfortable with it”).

#### Perceived/actual barriers to psychotherapy

To assess barriers to psychotherapy, a 12 (no experience of psychotherapy)- or 19-item measure (experience of psychotherapy) was adapted from the Barriers to Mental Health Services Scale-revised (BMHSS-R)^41^. Participants with and without psychotherapy experience were asked how hindering certain psychotherapeutic care-related factors (e.g., healthcare entitlements, transportation to psychotherapist’s office, psychotherapist’s cultural background) would be to access psychotherapy, on a four-point Likert scale (from “not at all” to “a lot”). We calculated a composite mean score for barriers constructed of the mean score for each participant, based on their experience with mental healthcare services. We opted for available item analysis^42^ to address missing values following two checks: (1) missing values occurred at random (Little MCAR test, *p* = 0.25) and (2) including for analysis only participant responses with less than 50% missing items yielded similar model results. The internal consistency of both versions of the scale was the same and very good (α = 0.90) for the present study.

#### Social resources for psychotherapy

Participants responded to two questions to assess social resources around seeking psychotherapy: (1) “Do you know someone who can help you find a psychotherapist if you are having emotional difficulties?” and (2) “Has anyone ever told you to go to a psychotherapist?” with either “yes” or “no.”, for those who answered the question above.

### Statistical analyses

We first ran descriptive analyses between the study variables and sociodemographic variables. We then performed three regression models: (1) a binary logistic regression to examine the relative contribution of barriers to psychotherapy, internalizing symptoms, and externalizing symptoms to seeking help from mental health-related professionals among the participants who reported emotional difficulties, (2) a binary logistic regression to examine the relative contribution of age, internalizing symptoms, and externalizing symptoms to seeking help from people outside of the mental healthcare system, and (3) a multinomial logistic regression to examine the relative contribution of barriers to psychotherapy, reporting emotional difficulties, and social resources to seeking psychotherapy. The assumptions of binary logistic regression (e.g., a linear relationship between continuous independent variables and the logit transformation of the dependent variable as checked by Box–Tidwell test) and multinomial logistic regression (e.g., collinearity check through Tolerance and VIF) were checked and fulfilled^43^. We opted for listwise deletions for regression models. All data analyses were performed with SPSS 26. The significance level was set at *p* < 0.05.

### Qualitative data collection and analysis

To gain a better understanding of the contextual factors influencing the mental healthcare access and use of ASR adolescents in Germany, we (AF and DP, who are public health researchers with prior experience in qualitative research) interviewed a variety of mental health professionals (i.e., school psychologists, psychotherapists, social workers, and pedagogues).

Following a situational analysis framework^44,45^, we naively approached the situation we defined as *ASR adolescents’ access to mental healthcare in Germany*. We investigated how different worlds—universes of discourses and actors committed to a common goal or agenda—contribute to the broader ecology of mental healthcare provision for ASR adolescents in Germany. The mental healthcare provision world comprises mental health service providers for minors (e.g., child and adolescent psychotherapists, psychiatrists, school counselors, and school psychologists) whose main commitments are identifying, assessing, and treating mental health issues among children and adolescents (ASR adolescents included). We conducted semi-structured interviews with four child psychotherapists providing mental healthcare, a social worker/pedagogue, and four school psychologists. We recruited our interview partners after an initial mapping of mental healthcare providers in one of three study settings (i.e., six districts in a Northern German state) and then via word-of-mouth referrals from our interviewees (i.e., snowball sampling). All interview partners were provided with an information sheet describing the goals of the research to ensure informed consent.

We asked our interview partners the following common questions: (1) Which factors enable ASR adolescents’ access to mental healthcare/psychotherapy services?; (2) Which access barriers, if any, exist?; (3) How can ASR adolescents’ access to mental healthcare/psychotherapy services be improved? Prior to each interview, we reflected on our assumptions about the interview partner and adapted our interview guide accordingly.

The interviews were conducted in German, both in-person, in participants’ place of work (*n* = 4) and online (*n* = 5), lasted between 40 and 78 min, and were audio-recorded and transcribed. Following situational analysis guidelines, we (AF and DP) performed open and axial coding, and memo writing^45^. We conducted constant comparative analysis through mapping, coding, and reflexive memo writing as a team (AF, DP, and YN)^46^, focusing on ongoing theorizing of the generated data. Data were analyzed using MAXQDA 2020^47^. While the situational maps are presented elsewhere^48^, we present findings from our interviews as they contextualize the quantitative findings. Interview quotes were translated into English and edited for clarity. In the results section, qualitative results are presented alongside quantitative results to support and contextualize each finding.

### Reporting summary

Further information on research design is available in the Nature Research Reporting Summary linked to this article.

## Results

### Sample demographic characteristics

Data from adolescents between 11 and 18 years of age coming mainly from Syria, Afghanistan, and Iraq, who arrived in Germany after 2015 were analyzed. Our subsample consisted of *N* = 216 participants who filled out the mental healthcare-related questions of the baseline sample, following exclusion based on country of origin, age, year of arrival, and procedural issues regarding the data collection process. Only participants who answered the question—“Do you think you have emotional difficulties that you need help with?”—were included in the analysis. The sociodemographic characteristics are presented in Table 2.

**Table 2:** Sociodemographic variables (*N* = 216).

Mean age (valid responses = 197)	M (SD) 14.66 (1.84)
Age in years	*n* (%)
11–12	28 (14.2)
13–14	73 (37.1)
15–16	55 (27.9)
17–18	41 (20.8)
Gender (valid responses = 211)	*n* (%)
Male	111 (52.6)
Female	98 (46.4)
Other	2 (1)
Living with parents (valid responses = 181)	*n* (%)
Yes	175 (96.7)
No	6 (3.3)
Country of origin (valid responses = 216)	*n* (%)
Syria	101 (46.7)
Iraq	54 (25)
Afghanistan	25 (11.6)
Other or unclear	36 (16.7)
Native language (valid responses = 205)	*n* (%)
Kurmancî (Northern Kurdish)	86 (42)
Arabic	74 (36.1)
Dari	17 (8.3)
Farsi	10 (4.9)
Sorani (Southern Kurdish)	10 (4.9)
Other	8 (3.9)
Reason for leaving country of origin (more than one answer possible)	*n* (%)
Conflict or war	170 (78.7)
Poor living conditions	26 (12)
Lack of prospects or poverty	12 (5.6)
Discrimination on grounds of religion	13 (6)
Discrimination on grounds of political activity	10 (4.6)
Discrimination on grounds of ethnicity	6 (2.8)
Other	5 (2.3)

### Reported emotional difficulties and psychotherapy utilization

Out of the 216 participants who answered the question, 65 (30.1%) reported having emotional difficulties for which they needed help. There were no gender or age differences in reporting emotional difficulties and no differences based on the study site.

Regarding mental health symptoms, 12.3% of our sample scored above the clinical cutoff (>69 points) on HSCL-37A. The mean scores were 15.18 (SD = 2.97) for externalizing symptoms and 39.80 (SD = 9.57) for internalizing symptoms.

The psychotherapists we interviewed further informed us that their ASR adolescent clients witnessed trauma, often accompanied by flight-related injuries. They believed trauma-related issues to be underdiagnosed within the mental healthcare system. They were further concerned that more recent arrivals in Germany (compared to the initial movement of 2015) had experienced more stressful life events and torture due to changes in the migration routes.

A little over half of our sample (53.2%) knew someone who could help them find a psychotherapist for their emotional difficulties. Someone in their lives (i.e., parent, relative, friend, religious mentor, and compatriot) or a professional (i.e., social worker, physician, and teacher) told 13.4% of the responding participants that they should go to a psychotherapist. However, only 6.9% had been to and 4.3% intended to go to a psychotherapist.

Out of the participants who reported emotional difficulties (*n* = 65), ten (15.4%) had been to a psychotherapist and seven (10.8%) intended to go to one. In our entire sample, 15 had been to a psychotherapist overall. Eleven were satisfied with the treatment they received. An interpreter accompanied eight of the participants in psychotherapy sessions. All found that the interpretation was done well and five found the interpreter trustworthy.

Three participants were still in psychotherapy. Those who quit psychotherapy did so for various reasons (multiple answers were possible): feeling better (*n* = 6), not feeling comfortable with the psychotherapist anymore (*n* = 7), not seeing the same psychotherapist at every visit (*n* = 2), being discouraged by friends or family (*n* = 2), other ways of coping suggested by a religious leader (*n* = 2), thinking that psychotherapy was not helpful (*n* = 1), difficulties finding suitable transport to get to the psychotherapist’s office (*n* = 1), and not receiving a call back from the psychotherapist despite wanting to go (*n* = 1).

Our interview partners provided context to the underutilization and non-continuation of services. They emphasized that accessing mental healthcare services are difficult for everyone in Germany, especially children and adolescents, but that ASR face additional challenges in navigating the mental healthcare system. They reported initial treatment delays in 2015/2016 while an allocation and support structure was being established. Now that this exists, our interview partners reported administrative problems as significant barriers to service provision. For example, statutory health insurance covers only around ten sessions, and only those that take place get paid. Since ASR clients lack stability, are often relocated, and therefore cannot regularly attend psychotherapy sessions, fears of income loss in case of canceled sessions act as negative incentives for psychotherapists.

Similarly, there were unclarities regarding who is in charge of organizing interpreters (the psychotherapist or the client) and who pays for their services. Although interpreter pools are covered by so-called “integration funds” in some cities, this is not standard practice. Uncertainties regarding ASR adolescents’ age or asylum status further complicate service provision. ASR adolescents’ legal status and the associated health entitlements were other significant barriers. One interview partner who treats torture survivors said that if her clients had a secure status, they would be able to benefit from regular care and would have been treated in inpatient care (as needed) rather than struggling with outpatient services. For many, the right to stay in Germany is dependent on being in school or apprenticeship, which makes getting the needed treatment nearly impossible: “*My clients, if they were German, they would all be treated in inpatient settings…. That’s simply [not possible] because of their status and because we can’t say to a refugee, “we’re going to put you in therapy or inpatient therapy or rehabilitation for two months,”- that would be a ridiculous recommendation to give* [because they have to stay in apprenticeship /school, otherwise they lose their residence permit and risk deportation]”.

Overall, services were insufficient in number and lacking in staff and funding, leading to significant waitlists (i.e., more than six months) on the ASR adolescents’ side and being overworked on the psychotherapists’ side. One interview partner reported that her office was the only provider in her region to offer psychotherapy to ASR adolescents and that they were fully booked for the whole year. This sole provider status also meant that they could not refer people on the waitlist to any other services. Furthermore, announced funding cuts to organizations serving migrants created further uncertainty.

Coordination difficulties were also reported. For example, one psychotherapist indicated that it was not always possible to give session appointments during school hours. This reflected a disagreement among the interview partners: the school psychologists highlighted that there was too much focus on trauma and psychotherapy when it comes to ASR minors’ well-being and that the school setting was an important stabilizing factor. As one school psychologist interview partner put it: *“They [teachers and schools] can calm, provide structure, routines, daily life, rituals, security; other children can be, so to speak, distractors, learning can be a distractor. So I think it’s more important to strengthen them and to see what therapeutic aids they bring with them, which also play into the therapist’s hands; the child is stabilized. So this very large area of clinicians, therapists, child, and adolescent psychiatrists in the context of trauma is not so, I don’t want to say relevant, but it is not such a big issue for us because it is simply not low-threshold and much too small and much too confusing”*.

Referral sources varied from pediatricians to youth services, but the interview partners thought that all different referral sources needed additional training in the mental health needs of ASR minors.

### Predictors of help-seeking patterns

#### Predictors of seeking help from mental health-related professionals

A binary logistic regression was run to assess the relative contribution of barriers to psychotherapy, internalizing symptoms, and externalizing symptoms to seeking help from mental health-related professionals among the participants who reported emotional difficulties. Scale characteristics are presented in Table 3.

**Table 3:** Scale properties across independent variables—Seeking help from mental health-related professionals.

	Not seeking help from mental health-related professionals (*n* = 35)*		Seeking help from mental health-related professionals (*n* = 14)*
Barriers	M(SD)	1.70 (0.55)	2.06 (0.55)
Externalizing symptoms	M(SD)	15.44 (2.60)	17.14 (3.32)
Internalizing symptoms	M(SD)	43.67 (10.70)	44.32 (9.26)

*N Includes only those who answered yes to having emotional difficulties for which they would need help.

The logistic regression model was statistically significant [χ2(3, *N* = 49) = 9.23, *p* < 0.05]. The model explained 25% (Nagelkerke *R*^2^) of seeking help from mental health-related professionals and correctly classified 89.8% of cases.

Externalizing symptoms and barriers to psychotherapy significantly predicted seeking help from mental health-related professionals: Participants with higher perceived or experienced barriers were more likely to have sought help from mental health-related professionals in reference to those who have not [*Exp*ß = 3.56, 95% CI (1.06, 12.01), *p* < 0.05]. Participants with higher externalizing symptoms were also more likely to have sought help from mental health-related professionals in reference to those who have not [*Exp*ß = 1.38, 95% CI (1.03, 1.86), *p* < 0.05]. Internalizing symptoms was not a significant predictor.

#### Predictors of seeking help from people outside of the mental healthcare system

A binary logistic regression was run to assess the relative contribution of age, internalizing symptoms, and externalizing symptoms to seeking help from people outside of the mental healthcare system among the participants who reported emotional difficulties. Scale characteristics are presented in Table 4.

**Table 4:** Scale properties across independent variables—Seeking help from people outside of the mental healthcare system.

	Seeking help from people outside of the mental healthcare system (*n* = 28)*		Not seeking help from people outside of the mental healthcare system (*n* = 18)*
Age	M(SD)	14.36 (2.16)	15.72 (1.56)
Externalizing symptoms	M(SD)	15.26 (2.31)	17.01 (3.50)
Internalizing symptoms	M(SD)	44.62 (9.85)	43.46 (11.36)

*N Includes only those who answered yes to having emotional difficulties for which they would need help.

The logistic regression model was statistically significant [χ2(3, *N* = 46) = 17.12, *p* = 0.001]. The model explained 42% (Nagelkerke R^2^) of seeking help from people outside of the mental healthcare system and correctly classified 76.1% of cases. All variables significantly predicted seeking help from people outside of the mental healthcare system: Participants who are younger in age were more likely to have sought help from people outside of the mental healthcare system in reference to those who have not [*Exp*ß = 0.59, 95% CI (0.39, 0.89), *p* < 0.05]. Participants with higher internalizing symptoms were also more likely to have sought help from people outside of the mental healthcare system in reference to those who have not [*Exp*ß = 1.16, 95% CI (1.03, 1.30), *p* < 0.01]. On the other hand, participants with lower externalizing symptoms were more likely to have sought help from people outside of the mental healthcare system in reference to those who have not [*Exp*ß = 0.56, 95% CI (0.37, 0.86), *p* < 0.05].

#### Predictors of seeking psychotherapy

A multinomial logistic regression was run to assess the relative contribution of barriers to psychotherapy, reporting emotional difficulties, and psychotherapy-related social resources to seeking psychotherapy. The logistic regression model was statistically significant [χ2(8, *N* = 113) = 24.15, *p* = 0.001]. The model explained 25% (Nagelkerke R^2^) of seeking help from mental health-related professionals and correctly classified 87.1% of cases. Overall effects of independent variables estimated by likelihood ratio tests revealed that the statistically significant contribution to the dependent variable is from being told by someone that they needed to go to a psychotherapist [χ2(2, *N* = 134) = 7.16, *p* < 0.05], and reporting emotional difficulties [χ2(2, *N* = 134) = 10.86, *p* < 0.01]. Knowing a person who could help them find a psychotherapist and perceived or experienced barriers were not significant predictors in this model.

Participants had an increased likelihood of intending to go to psychotherapy when they reported experiencing emotional difficulties [*Exp*ß = 6.87, 95% CI (1.28, 36.80), *p* < 0.05]. Participants had an increased likelihood of actually going to psychotherapy when they were told by someone that they needed to go to a psychotherapist [*Exp*ß = 6.72, 95% CI (1.63, 27.70), *p* < 0.01].

Our interview partners’ insights partly support these findings. In addition to the barriers summarized in the previous section, the interview partners highlighted the lack of familiarity with existing services among ASR minors and their families as an important reason to not seek psychotherapy when needed. One school psychologist indicated that it was crucial to motivate and help ASR adolescents to go to psychotherapy. Interview partners also reported that older adolescents received less support due to the social support systems in Germany. Although this wasn’t a group covered in our study, interview partners indicated that once young ASR turn 18, most support structures cannot provide help anymore, leaving young ASR by themselves to navigate social structures in Germany.

## Discussion

The present study aimed to describe ASR adolescents’ mental health and service use, and identify the predictors of their mental health-related help-seeking patterns. Our results revealed underutilization of mental health services relative to the emotional difficulties reported. We also found that access barriers were experienced in seeking help from mental health-related professionals. Moreover, we found that different symptoms were related to different help-seeking patterns and that psychotherapy-related social resources, as well as reporting emotional difficulties were predictors of actual or intended psychotherapeutic service use.

Compared to a representative cohort study, conducted between 2014 and 2017 in Germany (KiGGS2), which identified 16.9% of 12- to 17-year-olds to be at risk for mental health problems by using the Strengths and Difficulties Questionnaire^49^, the reported emotional difficulties within our sample were almost twice as high (30.1%). Although comparability between the two measures as well as the two samples is limited, our study indicates a potential high need for mental healthcare among ASR adolescents. Since out of the participants who expressed emotional difficulties, only 15.4% had been to a psychotherapist, this need remains largely unmet among our study’s participants. Unmet needs in ASR populations, as well as adolescents, have been widely reporteds^33,50–53^, so our findings are in line with previous findings.

In terms of internalizing (M = 39.80; SD = 9.57) and externalizing symptoms (M = 15.18; SD = 2.97), our sample had slightly lower scores than URM and accompanied refugee minors (ARM) in a German study on both subscales. In Müller et al.’s sample, ARM had lower externalizing symptoms scores (M = 14.13; SD = 1.99) and higher internalizing scores (M = 45.7, SD = 10.62), whereas URM had the highest internalizing (M = 50.71; SD = 12.27) and slightly higher externalizing scores (M = 15.96; SD = 2.78)^18^. Scores among refugee minors in Belgium^40^ and the Netherlands^54^ were similar to ones found in Müller et al.’s study, whereas our sample scored similarly to the Dutch native children in terms of internalizing symptoms (M = 39.5; SD = 9.1). All refugee minors across the three studies had lower externalizing scores than the native Dutch children (M = 18.3; SD = 4.5). To the best of our knowledge, no study employed HSCL-37A to study emotional distress among native German children, so the comparison to this group was not possible. While experiences in the countries of origin (predominantly Western and Eastern African countries, Morocco, Afghanistan, and Syria in the two studies) and resettlement have an impact on the level of psychological distress, these studies confirm the predominance of withdrawal, anxiety, and depressive symptoms among ASR children and adolescents mentioned in studies from previous decades^55^. The somewhat lower scores observed in our sample might be explained by the fact that most of our respondents were accompanied minors, benefitting from family support. Furthermore, since most ASR adolescents in our study were already in school for quite some time (and already fluent in German), they might have improved stability and social support, compared to those in the two studies.

In our study, participants with higher externalizing symptoms were more likely to have sought help from mental health-related professionals such as physicians, psychotherapists, and social workers. On the other hand, participants with lower externalizing symptoms and higher internalizing symptoms were more likely to have sought help from people outside of the mental healthcare system, such as family members and religious leaders. Externalizing symptoms have a higher likelihood of being noticed by people interacting with ASR adolescents (such as in the school setting) and thus are eventually more likely to be referred to the mental healthcare system. For example, an early study found that teachers are more likely to refer students with externalizing symptoms (than internalizing symptoms) for mental health treatment, even if they didn’t perceive externalizing problems as requiring more referral^56^. Externalizing symptoms are also more likely to involve institutional response (such as from criminal justice systems)^57^. An incident at the time of writing this article involved a police officer in Germany killing a 16-year-old unaccompanied refugee, when a youth welfare facility called the police following the adolescent’s suicide attempt^58^.

The pattern in referrals also mirrors the pattern in research: there are fewer studies and, therefore, less evidence about the help-seeking behaviors of adolescents with externalizing symptoms than internalizing symptoms^59^. A recent meta-analysis further provides an explanation for these findings: seeking help from informal sources (outside of the mental healthcare system) was associated with lower/decreased externalizing symptoms^59^. As our data were cross-sectional, both explanations are possible: help from friends, family, and other trusted resources may have lowered the number of the frequency of externalizing symptoms, or those with fewer externalizing symptoms were more likely to be perceived as approachable for help, when co-occurring with higher internalizing symptoms.

Social resources played an important role in psychotherapy-seeking among ASR adolescents. The fact that ASR adolescents were more likely to intend to seek psychotherapy than not if they disclosed emotional difficulties and that they were more likely to actually go to psychotherapy if they were told by someone close to them to do so, suggest that the path to psychotherapeutic care for ASR adolescents is complicated and multi-step. Although perceiving/experiencing barriers to psychotherapy did not predict seeking psychotherapeutic care, more barriers were associated with seeking help from mental health-related professionals. When our interview partners’ insights regarding access barriers to mental healthcare are considered, it can be concluded that, at least for this particular sample, someone else’s recognition of the problem facilitated psychotherapeutic care. This is in line with several studies that indicate problem recognition to be the most important predictor of accessing care in children and adolescents^50,51,60^.

The ASR adolescents who received psychotherapeutic care expressed general satisfaction both with psychotherapists and interpreters. Some of them stopped psychotherapy due to feeling better, though a small number did so because they were discouraged by social connections or structural barriers (not having the same psychotherapist for all sessions and difficulties finding suitable transportation to get to the psychotherapist’s office). This highlights the importance of social resources in the continuation of mental healthcare, and the importance of everyday settings such as schools in promoting mental health literacy to reduce mental illness stigma and facilitate help-seeking^16^.

The generalizability of our results is limited to our specific study locations by the non-representative sampling method (convenience sample), a relatively small sample size, and the number of missing values, which contributed to wide confidence intervals across models. We depended on administrators/professionals for our recruitment in schools and refugee accommodations and some refused collaboration, citing concerns regarding discussing mental health issues with ASR minors or limited resources (especially in schools). This had an impact on the number of ASR children and adolescents we could reach. While participants enthusiastically engaged with the survey, missing values most likely occurred due to the high number of items and the technical issues associated with tablet self-assessment. Furthermore, our sample was highly heterogenous, especially regarding mental health issues and service experience. The unfamiliarity with the concept of psychotherapy, despite mitigation on our part by definitions, might have contributed to lower response rates. However, we believe that recruiting participants and collecting data in familiar spaces such as schools or accommodations and relying on professionals/administrators who were familiar to the participants were also a strength, as these allowed participants to feel safer and more comfortable. We further identify the mixed-method approach as a strength of the present study. Interviewing mental health professionals provided valuable insights into the contextual and structural factors impacting ASR adolescents’ access to mental healthcare.

While the self-report nature of the survey meant that we gained insight into ASR adolescents’ own perceptions and experiences, unfiltered by parental or adult perspectives, there were clear limitations to this approach. Specifically, despite our efforts to simplify the language of the items, we could not get insight into contextual factors related to the length of stay, legal status, or healthcare entitlements, as adolescents could not report on them. This meant that their answers to these questions were unreliable and, therefore, not used in our analysis. We relied on mental health professionals’ insight to contextualize our findings instead. Furthermore, asylum statistics since 2015 have shown that the vast majority of asylum applications from Syria and Iraq are approved, whereas applicants from Afghanistan have higher percentages of rejections or delayed decisions^61–64^. Since December 2016, the German government has been conducting deportations to Afghanistan, a practice that has been socially and politically criticized^62–64^. As most of our participants are from Syria (46.7%) and Iraq (25%), it is possible that the majority also received protection for a period of 1–3 years^65^, indicating a less precarious legal status. In terms of health insurance entitlements, the 15–18 months post-arrival waiting period would apply to all participants, according to the Asylum Seekers’ Benefits Act (in German: Asylbewerberleistungsgesetz (AsylbLG)). The extent to which access to (mental) healthcare would be eased by their inclusion in the statutory health insurance system or by their provision with EHC, depends on the year of arrival and location of resettlement within each German federal state^26,30,66^.

Our findings indicate that ASR minors have an awareness of their emotional and mental health issues and ask for the support of those around them, or of a mental health professional if someone helps them navigate the complex German health system. Our results also confirm the findings of previous studies conducted with ASR minors^67,68^ and adolescents in general^24^, showing that they tend to encounter multiple access barriers to specialized care. Our results support the calls for ecological frameworks that look beyond individual factors contributing to the mental health of ASR adolescents and address factors at the family, community, sociocultural and political levels^55,69–71^ and allow for a “cultural diversity of healing”^72^. As ASR communities seek different forms of healing^73^ and support, we recommend that health systems recognize friends, relatives, teachers, and other significant people in ASR adolescents’ lives as important sources of support and essential interlocutors between adolescents and mental healthcare services. We, therefore, recommend that referral information or service knowledge is made available to these interlocutors. We furthermore recommend that internalizing symptoms such as withdrawal and anxiety are recognized as emotional difficulties for which ASR adolescents may need mental healthcare. In addition, given that adolescents turn to other people in their lives for help, we also recommend support be made available for those providing care (e.g., teachers, friends) to ASR adolescents as they perform the crucial role of bridging mental health needs and care. Strengthening the capacity of existing support structures (e.g., through training and funding) would facilitate the integration of multiple perspectives and approaches to healing and well-being that better reflect ASR adolescents’ concerns^70^. Incorporating low-threshold mental health prevention measures into settings routinely used and forms of social activities, and building upon the strengths of ASR adolescents would reduce their dependence on scarce professional/formal services.

Our interview partners highlighted the need for widespread, accessible, and low-threshold mental health initiatives designed to help those working with this group (i.e., in schools, refugee accommodations, etc.) to better identify mental health issues among ASR minors and help them navigate the complex German mental health system. Furthermore, they recommended interventions that focus on improving understanding around mental health issues, normalizing these issues and seeking help, as well as demystifying different types of services that could increase the likelihood of ASR minors asking for support, thus receiving specialized care when needed^24^. They identified many different actors, institutions, and agencies, such as youth services, pediatricians, refugee services, and professional chambers of healthcare professionals, as important information sources and referral points. They further underlined the importance of consensus among these different actors and institutions regarding how mental health needs are identified and addressed. Most importantly, most stated that without stability and safety (granted by a refugee status and residence permit), such interventions would have limited benefits or even be unethical, highlighting a secure legal status as an essential prerequisite to good mental health(care).

## Supplementary information

Reporting Summary

## Data Availability

Data were confidential but available upon reasonable request from the corresponding author. Requests for materials should be addressed to Yudit Namer, yudit.namer@uni-bielefeld.de
